# Depression, Anxiety, Stress, and Suicidality Levels in Young Adults Increased Two Years into the COVID-19 Pandemic

**DOI:** 10.3390/ijerph20010339

**Published:** 2022-12-26

**Authors:** Teodora Matić, Peter Pregelj, Aleksander Sadikov, Polona Rus Prelog

**Affiliations:** 1 Artificial Intelligence Laboratory, Faculty of Computer and Information Science, University of Ljubljana, 1000 Ljubljana, Slovenia; 2 Centre for Clinical Psychiatry, University Psychiatric Clinic Ljubljana, 1260 Ljubljana, Slovenia; 3 Faculty of Medicine, University of Ljubljana, 1000 Ljubljana, Slovenia

**Keywords:** depression, anxiety, stress, suicidality, Omicron, young adults

## Abstract

Background. The severity of both the COVID-19 clinical picture and confinement measures in Slovenia was higher during the initial phase of the pandemic in 2020 than during the Omicron wave in 2022. This could lead us to expect a higher level of distress during the initial phase. On the other hand, prolonged stress can have a detrimental effect on mental health. This study aimed to explore how the prolonged stress of the COVID-19 pandemic and the accompanying changes affected the mental health of young adults in Slovenia. We analyzed and compared the levels of depression, anxiety, stress, and suicidal ideation in young adults during the initial phase of the pandemic and the Omicron wave, as well as between the COVID-19-infected and non-infected individuals. Methods. An online survey was used to survey 587 young adults in the first wave (July–December 2020) and 511 in the Omicron wave (January–February 2022). Levels of depression, anxiety, stress, and suicidal ideation were compared using Mann–Whitney U test. Results. Results show that the Omicron wave significantly worsened depression, anxiety, stress, and suicidal ideation. Young adults who had tested positive for COVID-19 reported no worse or only slightly worse mental health than those who never tested positive. Conclusions. The current study provides new evidence about the mental health of young adults during the Omicron wave. Our results show that two years into the pandemic, they expressed more negative emotions and suicidal thoughts than at the beginning.

## 1. Introduction

The spread of severe acute respiratory syndrome coronavirus 2 (SARS-CoV-2) causing the coronavirus disease 2019 (COVID-19) pandemic has resulted in an array of negative effects on many aspects of societal functioning and has affected the physical and mental health of the population across the world [1]. Almost all countries have adopted confinement measures, including lockdowns, home isolation, and physical distancing. 

In November 2021, the World Health Organization (WHO) designated B.1.1.529 as a variant of concern named Omicron [2]. Highly infectious and more resistant to the existing vaccines, the Omicron variant led to a new surge in the number of infections and, at least at the beginning of the wave, new confinement measures around the world [3].

A large number of surveys have been carried out to determine the impact of the pandemic on mental health [4,5]. The importance of the viral infection with SARS-CoV-2 and pandemic-related containment measures on the mental health of the general population were studied across the world, mostly in the early phases of the pandemic [6,7,8,9]. 

Even though the clinical picture of the COVID-19 disease was considerably milder in younger than in older people in all the variants of the disease, younger people have almost uniformly shown to be disproportionally affected by mental health consequences of the pandemic [6,7,8]. 

Recent studies have demonstrated that young adults’ anxiety and depression are widespread and have risen significantly from pre- to during COVID years (from 2000–2019 to 2020–2022) [10,11]. Younger adults aged 18–39 years even experienced greater increases in higher levels of anxiety symptoms (from 9% to 21%) and moderate to high depression symptoms (9–39%) than any other adult age group [5,11,12,13,14]. A United Kingdom study during the first lockdown in the Spring of 2020 also found that suicidal ideation had increased, especially among young adults [15,16]. Thus far, less is known about how these symptoms (DAS) evolve over time.

Among the factors that have contributed to stress-related anxiety and depression in young adults, confinement measures that pronounced the challenges in employment, financial instability, and limited social interactions have been proposed [17,18,19].

In contrast, some authors argue that the viral infection itself might play a more important role in the etiology of depression and anxiety. Several studies have demonstrated [20,21] that infection with COVID-19 can be followed by heightened depression and anxiety symptoms [22,23].

The severity of both the COVID-19 disease/clinical picture and the following lockdown and confinement measures in Slovenia were considerably higher during the initial phase of the pandemic in 2020 than during the Omicron wave in 2022. This could lead us to expect a higher level of distress during the initial phase of the pandemic. On the other hand, prolonged stress has also been shown to have a detrimental effect on mental health [24]. However, the majority of studies were conducted during the first year of the COVID-19 pandemic, and the data on the mental health of younger adults during the subsequent waves, especially the recent Omicron wave, are still scarce. Despite the possibility that the effects of the COVID-19 pandemic on mental health have changed over time, the longstanding effects of the pandemic on young adults have been far less studied. Furthermore, there is a lack of studies on this topic conducted in Slovenia. Knowledge of nation-specific factors for mental health decline in youth is essential for mental health service and prevention planning. To the best of our knowledge, no studies have examined the mental health of young adults during the Omicron wave in Slovenia. Both the infection-related and pandemic-stress-related findings could lead us to expect mental health to deteriorate or resolve/improve throughout the course of the pandemic due to the milder course/clinical picture of the infection but, on the other hand, faster spread of the infection and longer duration of the pandemic and related circumstances.

In view of the above, the goal of this study was to explore the mental health changes in the younger population during the Omicron wave. To test these hypotheses, we analyzed and compared the levels of depression, anxiety, stress (DAS), and suicidal ideation in young adults during the initial phase of the pandemic and the Omicron wave. We also tested for differences in the above-mentioned measures between the respondents who had tested positive for COVID-19 at any point of the pandemic and those who had not, to disentangle the possible effects of the COVID-19 infection from the effects of the overall stress caused by the epidemiological situation and its social and economic implications.

## 2. Materials and Methods

### 2.1. Study Design and Participants

This population-based study was a part of a large international multicenter study that started in Italy during the first wave of the pandemic [25]. We used the study protocol questionnaire adapted for the Slovenian population. An online survey was implemented through a multistep procedure: (a) email invitation to healthcare professionals through their institutions, (b) social media channels (Facebook, LinkedIn) with snowball sampling strategy, (c) mailing lists of universities, and (d) other official websites or mailing lists (e.g., healthcare or welfare authorities’ websites, companies, etc.). The survey took approximately 20 min to complete. The study was approved by the Republic of Slovenia National Medical Ethics Committee under protocol no. 0120-283/2020/7.

The first wave of data was collected from 23 July to 31 December 2020. The Omicron (mostly connected with the Omicron SARS-Cov2 variant) wave of data was collected from 1 January to 23 February 2022. In both waves, data collection, as described above, was performed in the nationwide community sample of the Slovenian adult population. The total sample collected in the first wave was 1785, while the total sample in the Omicron wave was 1241.

The present study focused on young adults, defined as 18–32 years old. While there is no universal definition of “young adults”, after a search of various United Nations bodies and committees, we chose the upper threshold of 32 as a good compromise in terms of the sample size and balance between subsets (defined later). In the first wave, there were 587 respondents in this category, while in the Omicron wave, there were 511.

### 2.2. Assessment Tools

Demographic variables analyzed included age, marital status, education, number of people in the household, number of children, presence of elderly or disabled, presence of physical or mental illness, and COVID-19 circumstances (presented in Table 1). In addition, an extensive battery of instruments was used in the survey. Only those relevant to this study are presented here. 

The emotional states of depression, anxiety, and stress were assessed using the Depression, Anxiety, and Stress Scale—21 Items (DASS-21), which is a set of three self-report scales and a valid tool in assessing mental health in the general population [26]. The scores for each subscale can be translated into categories ranging from normal to extremely severe. Reliability of the DASS subscales in the young subpopulation was very good, with the Cronbach alpha for depression being 0.89 and 0.92, for anxiety 0.84 and 0.87, and for stress 0.92 and 0.92, in the first and the Omicron wave, respectively.

The Suicidal Ideation Attributes Scale (SIDAS) consists of five items assessing the frequency of suicidal thoughts, controllability, closeness to attempt, level of associated distress, and interference with daily functioning over the past month. Each item is assessed on a 10-level Likert scale, with a total score ranging from 0 to 50. In the case of scoring “0—Never” on the first item, all other items are skipped, and the total score is zero. The presence of any suicidal ideation is considered indicative of a risk for suicidal behavior [27]. The reliability of SIDAS in the young subpopulation was 0.87 in the first and 0.91 in the Omicron wave (Cronbach alpha).

### 2.3. Statistical Analysis

All comparisons between waves and subgroups were performed using the Mann–Whitney U test. We chose this non-parametric test due to a positive skew of all scores.

## 3. Results

### 3.1. Descriptive Statistics

There were in total 1098 young adults, 587 in the first and 511 in the Omicron wave. The average age of the young adults was 26.9 years in the first wave, with 77.0% being women, and 23.8 years in the Omicron wave, with 78.3% being women. There were 24.5% and 64.0% students, and 70.2% and 31.7% employed in the first and the Omicron wave, respectively. The students and the employed were non-overlapping categories due to how the questionnaire was structured. A higher proportion of students in the Omicron wave is the result of students being more directly targeted during the Omicron wave. The focus of data collection was on young adults, since our previous first-wave data analysis [14] showed that they were most at risk for mental health decline during the pandemic. Additional characteristics describing the sample are given in Table 1. Furthermore, an overview of the scores on the DASS and SIDAS scales is given in Table 2, while an overview of distribution across categories of DASS and SIDAS scores is given in Table 3.

### 3.2. Comparison between Waves

**Figure 1:**
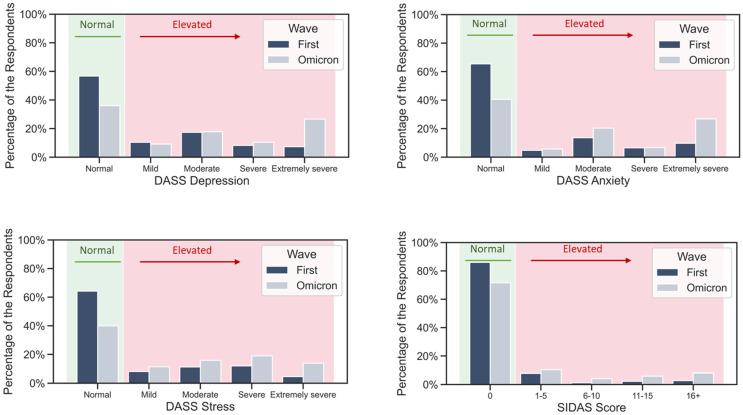
Distribution of DASS Depression, DASS Anxiety, DASS Stress, and SIDAS scores by category in the first and the Omicron wave, in the total sample. All the scores markedly increased in the Omicron wave.

Scores on the Depression, Anxiety, and Stress subscales of DASS, and total score on the SIDAS scale were compared between waves. For all four tested scores, the results were significant (*p* < 0.001), indicating higher scores in the Omicron wave. The distribution of the scales’ scores in both waves is shown in Figure 1. An increase in scores was found in both men and women.

**Figure 2:**
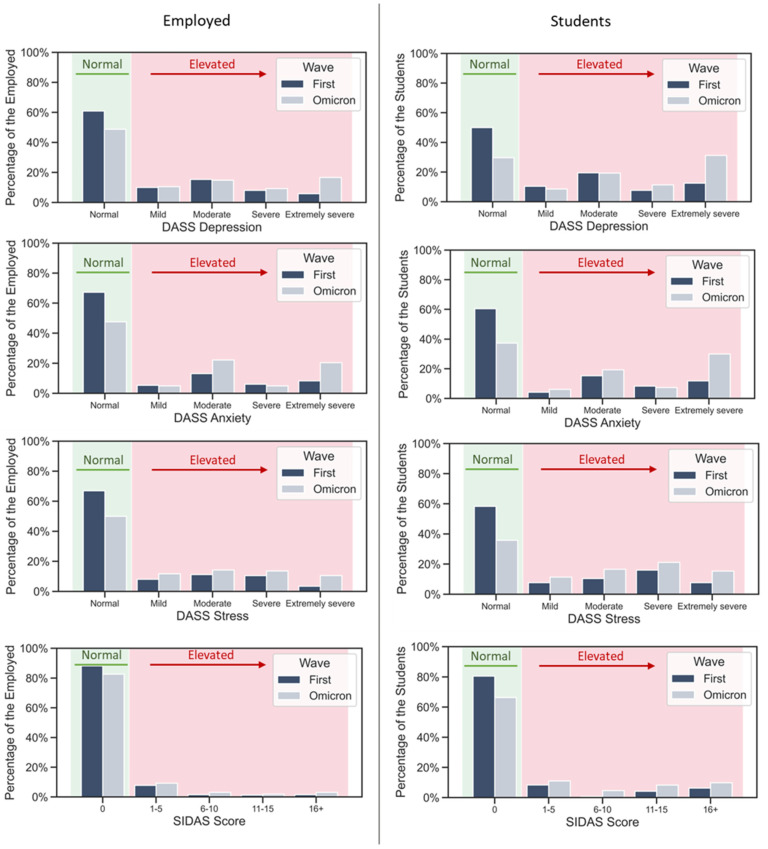
Distribution of DASS Depression, DASS Anxiety, DASS Stress, and SIDAS scores by category in the first and the Omicron wave, among the employed respondents and the students. Both employed respondents and students exhibited the same trend of increased scores in the Omicron wave.

Because of the different proportions of students and employed respondents in the two samples, we repeated the analysis separately on both groups and obtained the same result for all four scales and both groups. This lifestyle variable is also connected to other differences, e.g., educational differences, marital status, etc. The distributions of the scores for students and employed respondents are in Figure 2.

We also compared the proportion of the respondents who scored high (above 0) on SIDAS between the waves, using the Z test for proportions. With 14.0% of respondents scoring high in the first wave and 28.0% scoring high in the Omicron wave, the difference was highly significant (*p* < 0.001).

### 3.3. Comparison between the Respondents Who Had Tested Positive for COVID-19 and the Respondents Who Had Not

Scores on the Depression, Anxiety, and Stress subscales of DASS, and total score on the SIDAS scale were further compared between the respondents who had tested positive for COVID-19 and those who had not (who either had not been tested or had always tested negative). Due to the small fraction of respondents who tested positive for COVID-19 in the first wave, we used only data from the Omicron wave, in which 48.3% of the respondents received a positive test result at some point.

**Figure 3:**
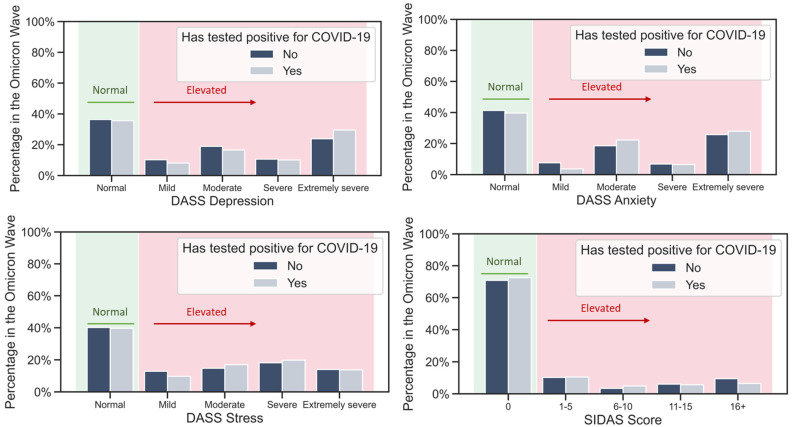
Distribution of DASS Depression, DASS Anxiety, DASS Stress, and SIDAS scores by category in the Omicron wave, among the respondents who had and the respondents who had not tested positive for COVID-19. The lack of differences between distributions indicates that a positive test for COVID-19 was not the reason for the observed increased scores in all the categories.

As the sample size is relatively large, we obtained significant *p* values for all scales: the DASS subscales (*p* < 0.001 for all three subscales, respectively) and SIDAS (*p* = 0.017). However, an inspection of the scales’ distributions (Figure 3) reveals that, while there is an overall trend of scores being slightly higher for the respondents who tested positive for COVID-19, the difference is far from striking, and it is especially small for the SIDAS score. 

### 3.4. Comparison of Respondents Who Did Not Test Positive for COVID-19 between the First and the Omicron Wave

To further assess the effect of the overall situational stress, we compared the subpopulation of the respondents who had not tested positive for COVID-19 between the waves on all scales.

**Figure 4:**
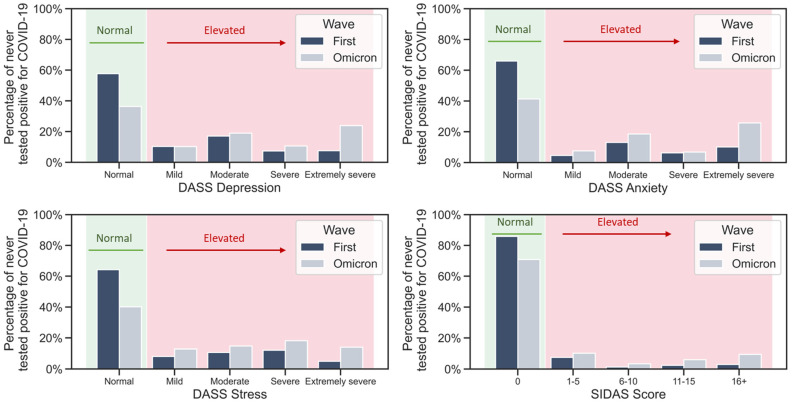
Distribution of DASS Depression, DASS Anxiety, DASS Stress, and SIDAS scores by category in the first and the Omicron wave, among the respondents who had never tested positive for COVID-19. The same increase can be observed in the total sample of respondents.

The results suggest a highly significant (*p* < 0.001) increase for all scales, evident in Figure 4. The DASS Depression score median increased from 6 in the first wave to 14 in the Omicron wave; the DASS Anxiety score median from 4 to 10; the DASS Stress median from 10 to 18; and the SIDAS median remained 0.

## 4. Discussion

### 4.1. Main Findings

This study analyzed and compared the levels of depression, anxiety, stress (DAS), and suicidal ideation in young adults during the initial phase of the pandemic and the Omicron wave. With validated and reliable assessment instruments, we investigated the trends in the most important domains of mental health in the youth. Our results show that the Omicron wave brought a significant worsening of DAS and suicidal ideation in the younger population. Even though the DAS levels were already high in the first wave of the COVID-19 pandemic in Slovenia, all the measured mental health parameters were significantly worse during the Omicron wave for both, women and men. Another objective was to evaluate the difference in DAS between the COVID-19-infected and non-infected young individuals. The young who tested positive for SARS-CoV-2/COVID-19 reported no worse or only slightly worse mental health parameters than those who never tested positive.

### 4.2. Previous Research and Possible Explanations

Through our two waves of data, we first observed the difference in DAS between the first wave and the Omicron wave in Slovenia. Our results show a significant increase in all scores for DAS and even for suicidal ideation. In line with the observation of other studies [17,28,29], our results highlight high levels of mental health distress experienced by young adults.

Medians show that during the first wave, most participants had normal levels of DAS, while in the Omicron wave, most participants reported at least moderate levels of depression and anxiety: only 36.0% had normal levels of depression, 40.5% had normal levels of anxiety and 39.9% normal levels of stress. Furthermore, 54.8% of the Omicron wave participants showed at least moderate levels of depression (moderate to extremely severe), while around a quarter reported extremely severe levels of depression and anxiety, which showed a greater increase than the stress symptoms. Over a period of two years, in our sample, the highest (extremely severe) levels of DAS were significantly higher for all three dimensions; extremely severe depression rose from 7.3% to 26.6% of participants, anxiety from 9.7% to 26.8%, and stress from 4.6% to 13.9%. Especially concerning is the significant rise in the reported level of suicidal ideation, from 14.0% in the first to 28.4% in the Omicron wave. 

Considering the known correlation between stress and depression and that it takes time for depression to evolve when the stress lasts, we can hypothesize that the increase might be the result of the prolonged stressful circumstances of the pandemic. The frequency of extremely severe depression and anxiety levels is especially concerning, as it suggests that at least some participants may have clinically significant depression.

The results of our study are difficult to compare since the literature on the mental health of the youth during the Omicron wave is still scarce. Among studies that used the same tool to evaluate DAS at the beginning of the pandemic, Wan Mohd Yunus et al. showed that university students scored moderate to extremely severe levels of DAS symptoms at 22.0%, 34.3%, and 37.3%, respectively, with levels of DAS significantly different according to age: younger students experienced more stress, anxiety, and depression symptoms compared with older ones [30]. Similarly, the prevalence of stress, anxiety, and depression among graduating class students was 22.2%, 39.6%, and 40.2%, respectively, in another study [31]. Comparable or even higher levels of DAS were found in the younger population of our sample during the first wave of data.

Even fewer studies observed the mental health of the youth in the subsequent waves of the pandemic. In line with the results of our study, a national repeated cross-sectional study in Norway showed that one year after the COVID-19 pandemic began, studying under prolonged restrictions may affect the lives of young adults, especially students [32]. Khan reported that 28.5% of the students experienced stress, 33.3% anxiety, and 46.9% depression from mild to extremely severe, according to DASS-21 [33]. 

Perhaps the most interesting finding of our study is that the difference in DAS was slightly more pronounced in those who never tested positive for SARS-CoV2. Even though studies show that viral infection with SARS-Cov2 with its immune inflammatory response and the subsequent potential neuroinflammation can be related to depression [23], along with psychological stressors induced by being infected [34,35], for our sample, psychological distress might be more important. These results are in line with a recent review that analyzed COVID-19 one year into the pandemic and found that the overall effect of the pandemic was linked with worsening psychiatric symptoms, while the long-term effect from direct COVID-19 infection was associated with no or mild symptoms [36]. It was also demonstrated that infection-related DAS has a short duration and is more prevalent in the severely ill and hospitalized [20], which could partially explain the results of our study. Considering that our study did not include information on the time of infection, the DAS symptoms may have already waned at the time of study participation, which is consistent with the short-term effect of the virus on mental health and in line with other etiological mechanisms for worse mental health. Similarly, a recent study on longitudinal data from seven European countries found no significant association between the onset of COVID-19 infection and changes in the probability of depression or anxiety [36,37,38,39]. 

Consistent with other studies and a recent systematic review, it could be more likely that indirect psychosocial factors may be the overriding mechanism for the increased level of anxiety and depression among the participants of our study that reported having had a COVID-19 infection [36,38,39]. Environmental factors such as intermittent quarantine and lockdown measures were shown to be risk factors for psychiatric disorders, along with psychological, social, and employment-related factors [14,40,41].

In the present study, two years into the pandemic, almost half of the Omicron wave participants had tested positive for SARS-Cov-2, and more than half had been in isolation. During the Omicron wave, infection rates in the general population in Slovenia were much higher and especially more prevalent among the younger population.

Although we cannot conclude the most defining factors for mental health deterioration from our study, there are other differences between the Omicron and first-wave samples. First, the Omicron sample is slightly younger (by 3 years), and many more are single (26.9 vs. 46.6%); however, the ratio of men and women is comparable. Second, 6.7% of participants lost a close person due to COVID-19 (0.3% in the first wave). Stress related to infection and the possibility of spreading it was also demonstrated to add to the burden of anxiety [40]. Lastly, during the Omicron wave, 13.8% of participants stated having been treated for a mental health problem, compared to only 6.8% during the first wave; however, the Omicron wave participants might have sought help due to the pandemic-related anxiety and depression. During the pandemic, more people in Slovenia, predominantly younger, sought psychiatric or psychological help, according to national data records. Our results are also consistent with a recent WHO review that suggests a worldwide increase in mental health problems, especially depression and anxiety in youth [42]. 

There might be several reasons for the youth’s mental health to deteriorate. Young adults were also often claimed as critical to limiting the virus’s spread. On the other hand, young adults often live in disadvantaged circumstances and are disproportionately unemployed, working in the informal economy, on precarious contracts, or in the service sectors that are more likely to be severely affected by COVID-19 [43]. 

#### 4.2.1. Strengths and Limitations

Ours is one of the few studies comparing DAS symptoms and suicidal ideation in the younger population at the beginning of the pandemic and during the Omicron wave. Strengths of our study include new data on depressive and anxiety symptoms during the Omicron wave and a comparison of the early and late stages of the pandemic. In addition, the number of respondents in both waves is well balanced and quite high, considering Slovenia’s total population of roughly 2.1 million people.

However, the findings of our study should be considered in light of its limitations. First, given the cross-sectional design of our study, a direct longitudinal comparison between the two waves cannot be drawn. Next, self-reported questionnaires can be influenced by biases such as memory errors and under-reporting. Additionally, convenience sampling means that the results are not necessarily generalizable to the general young adult population in Slovenia. Online convenience sampling may have also included more participants who were already more engaged and interested in the topic. On the other hand, since the whole questionnaire was quite long, the participants with poor concentration due to mental health problems may not have ended the questionnaire, and thus, our results may even be underestimated. It was also demonstrated that non-participants of health surveys generally have worse health than participants [44]. Next, the 13.5% who reported having mental health issues can also include some people with preexisting mental conditions, but these cannot represent such a big difference in the two samples. Finally, it is also likely that some of the participants in either wave had not had confirmed COVID-19 disease or had not been tested. 

#### 4.2.2. Clinical Implications

The finding that 40% of participants reported symptoms of anxiety and depression two years into the pandemic should induce response strategies. Along with preventing the spread of the disease, parallel mental health pandemics in the youth should be addressed as well. Prevention programs should be implemented to decrease stress and mitigate DAS in vulnerable groups, especially younger people. Concomitant stress seems to have an important role in the etiology; therefore, more actions must be directed at the preventive measures to reduce stress in the younger (such as relaxation-technique education, risk-factor awareness raising, coping strategies, meditation, mindfulness, and other techniques) and education of depressive and anxiety symptom recognition. Additionally, support groups can be provided for the most vulnerable. In the context of such widespread and prolonged changes, the numbers could add up to a substantial clinical burden. Adequate policies and mental health system capacities should be planned accordingly. 

## 5. Conclusions

The current study provides new evidence about the mental health status of young adults during the Omicron wave. Our results show that two years into the pandemic, young adults express more DAS and suicidal thoughts than at the beginning of the epidemic.

The findings from the current study add to the emerging evidence demonstrating a decline in mental health during the pandemic, which seems to be consistent across the globe. Younger adults may be more vulnerable to the mental health impact. The findings of our study suggest that pandemic circumstances and psychosocial factors may be more important than the impact of COVID-19 infection on the mental health of younger adults. Further research and the longitudinal following should be planned to disentangle these relationships. Preventive programs and mental health system capacities for the youth should be developed accordingly.

## Figures and Tables

**Table 1:** Demographic, lifestyle, and COVID-19-related characteristics of the sample, in the first and the Omicron wave.

		Wave 1	Wave 2
Marital status (distribution)	Married/Living with partner	52.6%	27.6%
	In a relationship and not living with partner	20.3%	25.6%
	Single	26.9%	46.6%
	Divorced	0.2%	0.2%
Employment status (distribution)	Students	24.5%	64.0%
	Employed	70.2%	31.7%
Education (distribution)	Ph.D./Specialization	4.4%	3.1%
	Master’s degree	27.0%	24.9%
	Graduate degree	29.0%	16.4%
	High school	29.5%	55.6%
	Elementary school	0.2%	0.0%
Number of people in the household	Mean (±SD)	3.1 (±1.5)	3.5 (±1.6)
Has children	% Yes	16.4	6.7
Has a physical illness	% Yes	10.2	9.2
Has a mental illness	% Yes	6.8	13.5
Has tested positive for COVID-19	% Yes	7.3	48.3
Has been hospitalized for COVID-19	% Yes	4.7	0.0
Has had pneumonia caused by COVID-19	% Yes	0.0	1.6
Has been in isolation due to COVID-19	% Yes	17.5	58.9
Has been in isolation due to contact with a person infected with COVID-19	% Yes	13.6	48.3
Close person has had COVID-19	% Yes	17.9	80.6
Close person died of COVID-19	% Yes	0.3	6.7
Pregnant women or newborn in the household	% Yes	6.6	4.3
Elderly or chronically ill person in the household	% Yes	22.8	25.2

**Table 2:** Mean (±the standard deviation from the mean) and median scores on DASS Depression, DASS Anxiety, DASS Stress, and SIDAS scales in the first and the Omicron wave, in the total sample.

		Wave 1	Wave 2
DASS Depression (0–42)	Mean (±SD)	10.1 (±9.6)	16.9 (±12.5)
	Median	8 (normal)	16 (moderate)
DASS Anxiety (0–42)	Mean (±SD)	6.7 (±7.9)	12.3 (±10.7)
	Median	4 (normal)	10 (moderate)
DASS Stress (0–42)	Mean (±SD)	12.5 (±10.7)	18.7 (±11.9)
	Median	10 (normal)	18 (mild)
SIDAS (0–50)	Mean (±SD)	1.2 (±4.5)	3.5 (±7.9)
	Median	0 (normal)	0 (normal)

**Table 3:** Distribution of DASS Depression, DASS Anxiety, DASS Stress, and SIDAS scores by category in the first and the Omicron wave, in the total sample.

		Wave 1 (%)	Wave 2 (%)
DASS Depression	Normal (0–9)	56.7	36.0
	Mild (10–12)	10.4	9.2
	Moderate (13–20)	17.4	17.8
	Severe (21–27)	8.2	10.4
	Extremely Severe (28–42)	7.3	26.6
DASS Anxiety	Normal (0–6)	65.4	40.5
	Mild (7–9)	4.8	5.7
	Moderate (10–14)	13.6	20.4
	Severe (15–19)	6.5	6.7
	Extremely Severe (20–42)	9.7	26.8
DASS Stress	Normal (0–10)	64.2	39.9
	Mild (11–18)	8.0	11.4
	Moderate (19–26)	11.2	15.9
	Severe (27–34)	11.9	19.8
	Extremely Severe (35–42)	4.6	13.9
SIDAS	Low (0)	86.0	71.6
	High (1 or more)	14.0	28.4

## Data Availability

Not applicable.
